# Thermal Analysis of High Entropy Rare Earth Oxides

**DOI:** 10.3390/ma13143141

**Published:** 2020-07-14

**Authors:** Sergey V. Ushakov, Shmuel Hayun, Weiping Gong, Alexandra Navrotsky

**Affiliations:** 1 School of Molecular Sciences, and Center for Materials of the Universe, Arizona State University, Tempe, AZ 85287, USA; 2 Department of Materials Engineering at the Ben-Gurion University of the Negev, Beer-Sheva 84105, Israel; 3 Guangdong Provincial Key Laboratory of Electronic Functional Materials and Devices, Huizhou University, Huizhou 516001, China

**Keywords:** high entropy oxides, rare earth oxides, laser melting, aerodynamic levitation, phase transition, melting, thermodynamics

## Abstract

Phase transformations in multicomponent rare earth sesquioxides were studied by splat quenching from the melt, high temperature differential thermal analysis and synchrotron X-ray diffraction on laser-heated samples. Three compositions were prepared by the solution combustion method: (La,Sm,Dy,Er,RE)_2_O_3_, where all oxides are in equimolar ratios and RE is Nd or Gd or Y. After annealing at 800 °C, all powders contained mainly a phase of C-type bixbyite structure. After laser melting, all samples were quenched in a single-phase monoclinic B-type structure. Thermal analysis indicated three reversible phase transitions in the range 1900–2400 °C, assigned as transformations into A, H, and X rare earth sesquioxides structure types. Unit cell volumes and volume changes on C-B, B-A, and H-X transformations were measured by X-ray diffraction and consistent with the trend in pure rare earth sesquioxides. The formation of single-phase solid solutions was predicted by Calphad calculations. The melting point was determined for the (La,Sm,Dy,Er,Nd)_2_O_3_ sample as 2456 ± 12 °C, which is higher than for any of constituent oxides. An increase in melting temperature is probably related to nonideal mixing in the solid and/or the melt and prompts future investigation of the liquidus surface in Sm_2_O_3_-Dy_2_O_3_, Sm_2_O_3_-Er_2_O_3_, and Dy_2_O_3_-Er_2_O_3_ systems.

## 1. Introduction

Alloys often contain tens of elements in strictly defined ratios with one element as “the base” of the alloy (e.g., all steels have more than 70 at.% Fe). Recently, a new design approach had emerged, which is focused on “baseless” or multi-principle element alloys (MPEAs) with the concentration of each element no more than 35 at.% but not less than 5 at.% [1]. The reports on complex, concentrated alloys (CCAs) appeared in the literature since the 1960s [2]; however, the new research direction took off in 2004 after the discovery of remarkable hardness, yield strength, and resistance to annealing softening in several MPEAs made by Taiwanese metallurgists [1,3]. They also introduced the term “high entropy alloys (HEA),” arguing that in these complex compositions, the gain in configurational entropy is responsible for the formation of simple single-phase solid solutions, rather than intermetallic compounds which would have a deleterious effect on the properties.

The high entropy design approach was recently applied to carbides [4,5] borides [6,7,8], and oxides [9,10,11,12,13,14,15,16,17,18] for high temperature and battery-related applications. Most of the high entropy compositions that were successfully prepared as single phases are within the 15% limit of atomic radii differences known as a Hume-Rothery [19] rule to metallurgists and as a Goldschmidt [20] limit for isomorphic mixtures to mineralogists (the majority of mineral species meet HE definition! [21]). While the argument about the role of configurational entropy is highly contentious [2,22], the name has its merits and rightfully attracts attention to thermodynamic controls, and we use the high entropy (HE) term to refer to five component rare earth oxides studied in this work.

**Figure 1:**
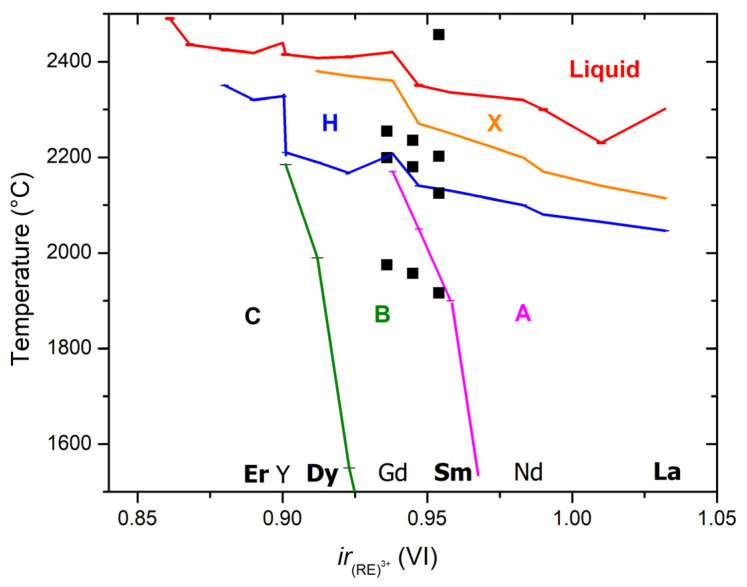
Phase transformations in rare earth and yttrium sesquioxides vs. ionic radii for octahedral coordination. Lines connect the best values for pure sesquioxides. The data points represent temperatures of phase transitions from thermal analysis of three (La_0.2_Sm_0.2_Dy_0.2_Er_0.2_RE_0.2_)_2_O_3_ compositions studied in this work, where RE–Nd, Gd, or Y, plotted vs. average ionic radius.

It soon will be a century since Goldschmidt et al. [23] published the first research on rich polymorphism in rare earth sesquioxides (R_2_O_3_, where R is a lanthanide, Y or Sc). They originally divided quenchable polymorphs into A, B, and C types (Figure 1). The A-type is trigonal (*P*-3*m*1), typical for sesquioxides of the large lanthanides, and also called La_2_O_3_-type; the B-type is monoclinic (*C*2*/m*), typical for lanthanides in the middle of series and also called Sm_2_O_3_-type [24]; the C-type is cubic (*Ia*-3), typical for small lanthanides, Y and Sc, and also called bixbyite-type after the naturally occurring (MnFe)_2_O_3_ mineral.

Most rare earth oxides can be obtained in more than one structure type (polymorph) at ambient conditions: normally an A-type La_2_O_3_ and Nd_2_O_3_ can be synthesized in a C-type structure [25] at temperatures below ~500 °C (and C-type was predicted [26] to be their ground state structure); while normally C-type oxides from Dy to Yb were obtained in B-type structure in nanoparticles [27]. The oxides of trivalent actinides also found in these structure types [28]. Two high temperature structures were first identified by Foex and Traverse [29,30]. The H-type is hexagonal (*P*63*/mmc*) [31] and was reported for all rare earth and Y sesquioxides except Lu and Yb [32]. For oxides from La to Dy, the transformation of the hexagonal phase to the cubic X-type (*Im*-3*m*) structure was detected before melting [33]. The X-type structure was also reported to be formed in Tm_2_O_3_ and Lu_2_O_3_ after irradiation with Xe and Au ion beams [34].

The systematic research on phase equilibria in rear earth oxides has mostly been focused on pure oxides and several binary systems. There are only a few systematic investigations of ternaries and they are limited to studies on quenched samples [35,36]. All the studied systems of trivalent rare earth oxides are characterized by wide ranges of solid solutions in the structures identified in pure oxides. Eleven interlanthanide perovskites are known to form in several systems combining large and small rare earths: LaRO_3_ (R = Y, Ho-Lu), CeRO_3_ (R = Tm-Lu), and PrRO_3_ (R = Yb-Lu) [37,38,39]. They all show an orthorhombic (*Pnma*) distortion and do not melt congruently, but decompose at 800–2000 °C into solid solutions of one of rear earth oxide structure types [39]. LaGdO_3_ in a B-type structure attracted attention for application as high-k gate dielectric [40] and as an optical temperature sensor when doped with Er/Yb [41,42].

Mixed three-four valent Ce, Tb, and Pr oxides with cubic defect fluorite related structures have been studied for gas sensor and catalyst applications [43]. Following the high entropy approach, Tseng et al. [44] studied thermal expansion and magnetic susceptibility of (Gd,Tb,Dy,Ho,Er)_2_O_3_ composition, which formed solid solution in a C-type structure. Djenadic et al. [11] reported that the presence of Ce^4+^ in several HE rare earth oxide compositions produced defect fluorite solid solutions.

In this work, we studied three compositions containing five rare earth sesquioxides in equiatomic ratios: (La,Sm,Dy,Er,RE)_2_O_3_, with RE either Nd or Gd or Y. All chosen rare earths are trivalent in the solid state, and their sesquioxides represent all polymorphs: A-type (La, Nd), B-type (Sm, Gd, Dy), and C-type (Er, Gd). However, they all form a H-type structure at high temperatures (Figure 1) with very intriguing properties, such as fast oxygen ion conductivity and superplasticity [45], but was never quenched to room temperature.

We performed laser melting, splat quenching, and annealing of the samples and characterized their high temperature phase transformations and thermal expansion by a combination of in situ differential thermal analysis and synchrotron diffraction on laser-heated samples. An unexpected and surprising finding was the substantial (>100 °C) increase in melting temperatures compared to those expected from consideration of melting points of constituent oxides.

## 2. Materials and Methods

The intimately mixed rare rear earth oxides of desired stoichiometry were first synthesized by the solution combustion method [46] and characterized by X-ray diffraction (XRD). Then, samples were laser melted in the hearth and in aerodynamic levitator and used for high temperature synchrotron XRD, differential thermal analysis (DTA), splat quenching, and prolonged annealing experiments. The experiment flow chart is provided in the Supplementary Materials (Figure S1).

### 2.1. Sample Synthesis

Aqueous solutions of rare earth nitrates (Sigma-Aldrich 99.9% metals base) were mixed in desired stoichiometry. Ethylene glycol and citric acid were mixed at a molar ratio of 1 to 2 and added to the nitrate water solution. The mixed nitrate–citrate solution was evaporated at 150 °C under agitation by magnetic stirring until a highly viscous foam-like colloid was formed. This colloid was annealed in air at 800 °C for 96 h. An additional heat treatment was performed at 1100 °C for 12 h. The samples were analyzed by room temperature powder X-ray diffraction after each treatment.

### 2.2. Laser Melting and Splat Quenching

Powders after heat treatment at 1100 °C were laser melted in air on the copper hearth with 400 W CO_2_ laser and remelted in an argon flow in the aerodynamic levitator. The resulting samples were oblate spheroids 2.6–2.9 mm in diameter, with a flattening of ~0.1. The structure and phase transformations in obtained samples were studied by XRD and DTA. Sample composition and homogeneity were characterized by electron microprobe analysis. Laser-melted spheroids were further processed by splat quenching using a splittable nozzle aerodynamic levitator. The employed device is part of a drop-and-catch (DnC) calorimeter, described in detail earlier [47]. For quenching experiments, solid copper plates were installed in place of the calorimeter sensors (Figure S2). The samples produced by splat quenching were analyzed by room temperature XRD.

### 2.3. Microprobe Analysis

A Cameca SX-100 electron microprobe was used for imaging and analysis of the chemical composition of laser-melted samples. Energy dispersive spectroscopy and backscattered electron imaging (BSE) were used for the characterization of sample homogeneity. Quantitative chemical analysis was performed by wavelength dispersive spectroscopy (WDS) using synthetic rare earth orthophosphate crystals for calibration standards for all rare earths except Y, for which synthetic Y_3_Al_5_O_12_ (YAG) was used due to flux originated Pb contamination detected in YPO_4_ standard.

### 2.4. Room Temperature X-ray Diffraction

Room temperature powder XRD was used to characterize samples after precipitation, laser melting, splat quenching, differential thermal analysis, and synchrotron diffraction experiments. The measurements were performed using Bruker D8 Advance diffractometer (Bruker, Madison, WI, USA) with CuK_a_ radiation and a rotating sample holder. The operating parameters were 40 kV and 40 mA, with a step size of 0.01° and dwell 3 s/step. Lattice parameters, phase fractions, and crystallite sizes of powders after annealing were refined using whole profile refinement as implemented in MDI Jade 2010 software package (Materials Data, Livermore, CA, USA). GSAS-II [48] was used for Rietveld [49] refinement of lattice parameters and phase fractions in laser-melted samples.

### 2.5. High Temperature X-ray Diffraction

High-temperature X-ray diffraction experiments were performed on an aerodynamic levitator at beamline 6-ID-D at the Advanced Photon Source (APS), Argonne National Laboratory. The levitator at the beamline provided by Materials Developments, Inc. (Evanston, IL, USA) and described in detail elsewhere [50]. The samples 63–70 mg in weight, were prepared by laser melting as described above.

The diffraction experiments were performed in transmission geometry with X-ray wavelength 0.1236 Å (100.3 keV energy). The beam was collimated in a “letterbox” shape, 500 µm wide and 200 µm tall. The samples were levitated in argon flow and heated from the top with a 400-W CO_2_ laser. The levitator software provided manual control of the levitation gas flow rate and manual or automated laser power control for sample heating. Diffraction data were collected in 100-°C increments based on the surface temperature of the levitated bead, which was monitored with a single color pyrometer (875–925 nm spectral band, IR-CAS3CS, Chino Co., Tokyo, Japan) with emissivity set to 0.92. Emissivities for rare earth oxides above 2000 °C are unknown [51], and thermal gradient in laser-heated aerodynamically levitated bead exceeds 100 °C [52,53,54]. In this work, the temperatures of diffracted volume were assigned based on phase transformation temperatures obtained from DTA measurements.

The diffraction images were recorded with a Perkin-Elmer XRD 1621 area detector positioned at a distance 1099 mm from the sample. The exposure time was set to 0.1 s to avoid detector saturation; 100 exposures were summed and recorded into a single image used for further processing with GSAS-II software [48]. The sample to detector distance, detector tilt, and beam center coordinates was calibrated using NIST CeO_2_ powder standard available at the beamline and with Y_2_O_3_ bead prepared by laser melting. The images from area detector were integrated from 1 to 7° 2Θ at 70–120 ° azimuth into diffraction patterns with 1600 points (0.00375 steps in 2Θ) (see Figure S3). Room temperature diffraction images were collected from every bead before and after laser heating. During the processing of diffraction data from the levitator, sample displacement was refined at room temperature from known cell parameters and kept constant during further refinements. Pawley [49,55] method, as implemented in GSAS-II, was used for refinement of unit cell parameters at high temperatures.

### 2.6. Differential Thermal Analysis

Differential thermal analysis was performed with a Setaram Setsys 2400 instrument modified to enable excursions to 2500 °C. The experiments were conducted in Ar flow at heating and cooling rates 20 °C/min using WRe differential heat flow sensor and thermocouple for furnace temperature control. 

Laser-melted beads, 100–140 mg in weight were placed in tungsten crucibles and sealed under Ar atmosphere to avoid the possibility of sample and standards contamination with carbon vapor from vitreous carbon protection tube. Multiple measurements were performed on each sample. Temperatures of phase transformations were determined as average from the onset [56] of endothermic peaks on heating. Enthalpies of phase transformation were calculated as the averages of absolute values of endothermic heat effects on heating and exothermic heat effects on cooling. The instrument and methodology were described in detail elsewhere [53,57,58,59]. Temperature and sensitivity calibrations were performed using melting and phase transition temperatures and enthalpies of Au (1064 °C), Al_2_O_3_ (2054 °C), Nd_2_O_3_ (A-H, H-X, and X-L at 2077, 2201 and 2308 °C, respectively), and Y_2_O_3_ (C-H and H-L at 2348 °C and 2439 °C, respectively). It must be noted that international temperature scale ITS-90 [60] defines no fixed points above the freezing point of gold, albeit alumina melting temperature 2054 ± 6 °C was recommended to be included as a secondary reference point on the ITS [61,62].

### 2.7. Calphad Modeling

Calphad [63,64,65,66] modeling was performed to compare with experimental results. Calphad-type thermodynamic database for rare earth sesquioxides was created by Zinkevich [67]. He critically reviewed all relevant experimental data available before 2006 and evaluated missing fusion enthalpies values based on measured enthalpy of fusion and volume change on melting for Y_2_O_3_. In a more recent evaluation by Zhang and Jung [68] evaluation, missing data were estimated from liquidus in RE_2_O_3_-Al_2_O_3_ phase diagrams. However, new measurements for fusion enthalpies [53] are in better agreement with Zinkevich’s assessment. Zhang and Jung’s evaluation varies largely with the values proposed by Konings et al. [69]. Zinkevich [67] database for rare earth sesquioxides is openly available at the NIST website [70] and was used in this work without any modifications. Thermo-Calc (Stockholm, Sweden) software was used for calculations.

## 3. Results

### 3.1. Chemical Composition

The composition was determined by microprobe analysis on laser-melted samples recovered after high temperature diffraction experiments. The measured ratios were close to nominal, indicating no preferential loss of any component on melting and laser heating during diffraction experiments. The microprobe results are reported in Table 1, with variations given as two standard deviations of the mean of 12 analyses per sample. Backscattered electron micrographs are included in Supplementary Materials (Figures S4–S6). The following stoichiometries of synthesized rare earth sesquioxides were obtained: (La_0.18_Sm_0.20_Dy_0.18_Er_0.18_Y_0.26_)_2_O_3_, (La_0.19_Sm_0.21_Dy_0.21_Er_0.20_Gd_0.19_)_2_O_3,_ and (La_0.20_Sm_0.20_Dy_0.21_Er_0.20_Nd_0.19_)_2_O_3_. For the sake of brevity, we will refer to these compositions as HE-Y, HE-Gd, and HE-Nd, respectively, where “HE” stands for “high entropy” and element symbol is for rare earth element in (La,Sm,Dy,Er,RE)_2_O_3_ nominal composition.

### 3.2. Phases after Solution Combustion Synthesis and Annealing

After 800 °C annealing of powders from solution combustion synthesis, the cubic C-type phase with crystallite size 20–40 nm was a major phase in all samples. The B-type phase was also detected in HE-Nd and HE-Gd samples (Table 2, Figures S7 and S8). After annealing of the powders at 1100 °C, only the B-type phase was identified in HE-Nd sample; the amount of B-type phase in HE-Gd sample increased to 70 wt.%. The C-type phase was retained in HE-Y composition, and its crystallite size increased to ~65 nm. The decrease in volume on C-B transformation was calculated from XRD data in HE-Nd and HE-Gd samples as 8% on average.

### 3.3. Phases after Laser Melting, Splat Quenching, and Annealing

**Figure 2:**
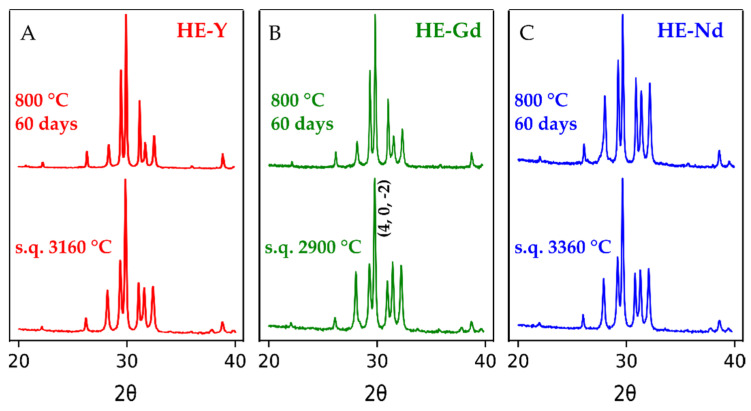
(**A**), (**B**), (**C**): powder X-ray diffraction patterns of three (La_0.2_Sm_0.2_Dy_0.2_Er_0.2_X_0.2_)_2_O_3_ compositions, where X–Y, Gd, or Nd, respectively. Patterns were collected at room temperature using CuK radiation (λ = 1.54056 Å) on samples obtained by splat quenching (s. q.) of the melts from indicated temperatures and after annealing at 800 °C for 60 days. All patterns identified as monoclinic (B-type) phases. Table 3 lists the results of cell parameters refinements. The typical profile refinement plot is presented in supporting information (Figure S9 in Supplementary Materials).

Melt processing yielded a B-type phase in all studied compositions. Room temperature XRD patterns are shown in Figure 2; the example of the whole profile refinement plot is included in Figure S9. The unit cell parameters and crystallite sizes of B-phase measured after splat quenching from ~3000 °C and after laser melting and 60 days annealing, are listed in Table 3.

In splat-quenching experiments, samples were heated in oxygen flow to the temperature several hundred degrees above the melting point to allow for cooling during ~100 ms drop time from the splittable nozzle to the splat-quenching plates. Sixty days annealing at 1100 °C was performed on the laser-melted samples, recovered after thermal analysis. The crystallite sizes of splat-quenched samples were about 80 nm. The crystallite sizes of the laser-melted samples after thermal analysis and annealing were in the range of 95–150 nm. The decrease in volume of B-type phase after annealing compared to splat-quenched samples was calculated from measured cell parameters as 0.6–0.8%. This variation is consistent with the retention of thermally induced defects in splat-quenched samples.

All samples after melting show an increase in the intensity of (4, 0, −2) peak of B-phase compare with calculated from an ideal B-type structure. This variation is not related to the complex composition of the studied sample since we observed a similar effect in pure Sm_2_O_3_ (Figure S10) and it is likely due to twinning [72].

### 3.4. Temperatures and Enthalpies of Phase Transformations from DTA Experiments

Thermal analysis was performed on laser-melted samples to enable sealing W crucibles; thus, the initial structure for all samples was B-type. The transition temperatures detected in the samples by differential thermal analysis are plotted in Figure 1; the data are summarized in Table 4.

On heating to 2400 °C, three reversible heat effects were observed in all samples, which were assigned to B-A, A-H, and H-X phase transformations (Figure S11). Enthalpies of transformations obtained from endothermic peaks on heating and exothermic peaks on cooling were generally consistent and were averaged to obtain the values listed in Table 4. The range of undercooling increased with transition temperature, with maximum observed values 14, 43, and 63 °C for B-A, A-H, and H-X transformations, respectively. For the A-H transition, the width of DTA peaks on heating in studied samples was similar to that observed for pure Nd_2_O_3_ at the same heating rate [59]. However, the peaks corresponding to B-A and H-X transformations were substantially wider than those for A-H in HE compositions and for H-X in pure Y_2_O_3_ and Nd_2_O_3_ (e.g., 41–44 °C for H-X transition in HE samples vs. 12 °C for pure Nd_2_O_3_).

Temperatures and enthalpies of transitions increased with decreasing average ionic radius, from HE-Nd to HE-Gd to HE-Y, consistent with the trend among pure rare earth sesquioxides. The exception was the enthalpy of B-A transition in HE-Y sample, which, although the same within calculated uncertainties, appeared ~2 kJ/mol smaller than that for B-A transition in HE-Gd sample.

Transition entropies were calculated from _trs_S = _trs_H/T, where T is the temperature in Kelvin at the transition onset on heating. Entropies of transitions are nearly constant between compositions, with average values ~9 J/mol/K for B-A transformation; ~3.4 J/mol/K for A-H transformation, and ~11.5 J/mol/K for H-X transformation, except for ~16 J/mol/K value for H-X transformation of HE-Y at 2254 °C. This deviation might be related to the fact that pure Y_2_O_3_ does not undergo H-X transformation.

**Figure 3:**
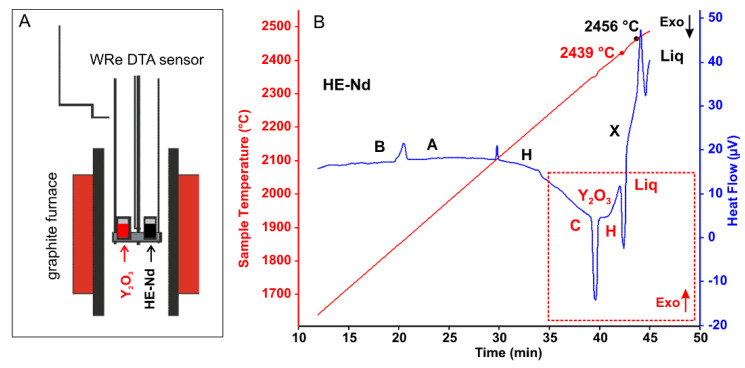
(**A**) The schematic of differential thermal analyzer and samples placement. (**B**) Heat flow trace (no baseline subtraction), showing heat effects on heating from HE-Nd ((La_0.20_Sm_0.20_Dy_0.21_Er_0.20_Nd_0.19_)_2_O_3_) and Y_2_O_3_ samples. Endothermic B-A-H-X-Liquid transformations for HE-Nd are labeled in black. Endothermic C-H-Liquid transformations for Y_2_O_2_ are labeled in red. The assignment of the exothermic direction of the heat flow signal for HE-Nd and Y_2_O_3_ is the opposite due to the placement of the samples. Melting temperatures for Y_2_O_3_ and HE-Nd are marked on the temperature trace. The Y_2_O_3_ melting point (2439 °C) was used for calibration.

During DTA experiments above 2300 °C, the failure of the sensor is frequent, and the maximum achievable temperature is limited by magnitude and direction of temperature drift of the control WRe thermocouple. For the calibration of the DTA thermocouple in this temperature range, pure Y_2_O_3_ (Tm 2439 ± 12 °C) [73] was used as a standard. All studied compositions were heated to temperatures above 2450 °C; however, the peak corresponding to melting onset was registered only for HE-Nd sample at temperature 17 °C above the melting point of Y_2_O_3_ (Figure 3). Due to the significant uncertainties in baseline choice and sensitivity calibration at this range, the enthalpy and entropy of fusion were not evaluated.

### 3.5. Volume Changes and Thermal Expansion from High-Temperature XRD

In diffraction experiments on levitated samples, powder-like diffraction patterns are obtained by ensuring the rotation of the solid sample in the gas flow [53,54,58]. When sample spheroids are prepared by melt solidification, as in this study, the exact shape of each bead depends on the surface tension of the particular composition, volume change on melting, and stochastic nature of nucleation. Due to these variations and crystal growth at high temperature, the rotation does not always produce the required random orientation of crystallites. In some cases, data are amenable to structure refinement [32,52]; however, in the present study, variations in intensities only allowed unambiguous identification of B-A and H-X transformations and refinement of the unit cell parameters of corresponding phases.

**Figure 4:**
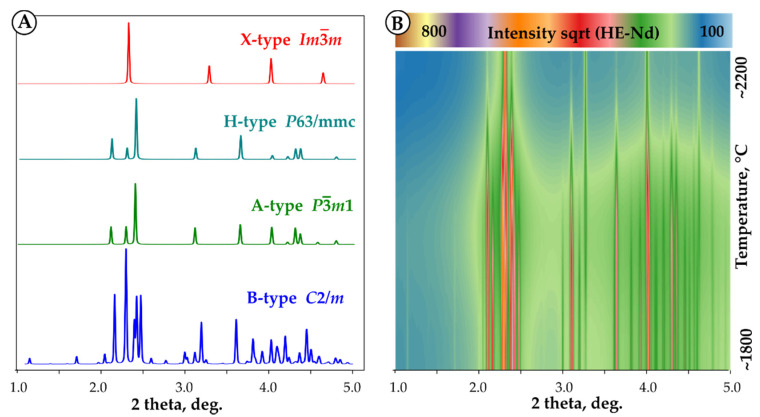
(**A**) Calculated X-ray diffraction patterns for different structure types for (La, Sm, Dy, Er, Nd)_2_O_3_ composition and instrument parameters corresponding to the experimental condition (λ = 0.1236 Å). Note the similarity of diffraction patterns for A and H structures. (**B**) GSAS-II contour plot of experimental diffraction patterns collected at 1800–2200 °C on laser-heated HE-Nd bead. See Figures S11 and S12 for refinement plots.

The A-H transformation shows well pronounced peaks in DTA measurements (Figure S11); however, the diffraction patterns of A and H phases are very similar (Figure 4). In our experiments, the data quality did not allow us to unambiguously identify the A-H transition from diffraction on levitated samples. Volume changes on B-A and H-X transformations were calculated from unit cell parameters at phase transformation temperatures. The data are presented in Table 5, and examples of refinements are included in Supplementary Materials (Figures S12 and S13). Both transitions are accompanied by volume contraction. The volume change on B-A transition was refined for all samples and found to be −2.5 ± 0.1% for HE-Nd and HE-Gd and −3.1 ± 0.1% for HE-Y (Table 5).

The thermal expansion of the B-type phase from room temperature to the B-A transition was calculated from room temperature cell parameters of annealed samples (Table 3) and cell parameters at the transition temperature (Table 5). The thermal expansion is anisotropic and similar for all three compositions. The smallest expansion is observed in *a* parameter (7.8 ± 0.7) × 10^−6^/K, linear thermal expansion coefficients along *b* and *c* directions are (1.6 ± 0.2) × 10^−5^/K and (1.2 ± 0.5) × 10^−5^/K, respectively. The average volume thermal expansion coefficient for all studied compositions in the B-type structure is (3.5 ± 0.2) × 10^−5^/K.

Due to the narrow temperature range of stability of A and H phases, the accurate calculation of thermal expansion is not possible. For HE-Nd sample, the average volume thermal expansion of A and H phases appears to be similar to that for the B phase, but for HE-Y an increase in thermal expansion is observed. The volume change on H-X transformation was refined for HE-Nd and HE-Y compositions as −1.2 ± 0.1% and −0.6 ± 0.1%, respectively.

## 4. Discussion

### 4.1. Experiment vs. Calphad Predictions

The Calphad approach is used widely in the high entropy alloys and ceramics field [74,75,76]. Senkov et al. demonstrated [74] that for high entropy alloys, Calphad computations of type and number of phases agreed well with experimental results only when more than half of binary systems were fully assessed; however, even when there are enough data on binary and ternary systems, Calphad predictions of transformation temperatures, phase compositions and fractions are challenging. Compared with metals, the databases for oxides are much more limited, especially for the temperature range addressed in this study. Nevertheless, it is instructive to compare our experimental results with Calphad predictions.

We used an open-access database created by Zinkevich [67]. It is based on his 2007 assessments of thermodynamic properties of pure rare earth sesquioxides from room temperature to the melting point. This database is often used as a starting point for the creation of new thermodynamic assessments for systems with rare earth oxides [77,78]. Without any modifications, the database allows modeling of phase equilibria using the ideal solution model (which assumes zero enthalpies of mixing and the largest configurational entropy gains). A set of interaction parameters for the regular solution model (which accounts for mixing enthalpies) are included in the Zinkevich review [67], but they are based on a limited number of selected binary systems, not included in the database [70] and were not used in the present study. The phase fractions as a function of temperature from Calphad modeling are shown in Figure S14.

#### 4.1.1. C-B Transition

After calcination at 800 °C, C-type was a major phase in all compositions and was the only phase detected in the HE-Y sample (Table 2). Calphad computations correctly predicted the formation of the C-type single-phase solid solution in HE-Y and as a major phase in HE-Gd and HE-Nd. After annealing at 1100 °C, a single-phase C-type solid solution was retained in the HE-Y sample, B-type solid solution became a major phase in He-Gd sample He-Nd completely transformed to B-type structure. The results for HE-Nd and HE-Gd are consistent with Calphad predictions; however, in HE-Y the B-type phase was predicted to appear but was not observed experimentally.

Laser melting and splat quenching produced B-type solid solutions in all samples. Calphad calculations predicted B-type single-phase field for all compositions, with low-temperature boundary shifting from 900 to 1400 °C with decreasing average ionic radius of RE from HE-Nd to HE-Y. The heating and cooling of laser-melted samples in DTA and two months of annealing at 800 °C did not reverse the transformation. The B-C transformation below 1000 °C is known to be kinetically hindered in pure oxides as well [79]; thus, retaining the B phase in our experiments does not indicate slower diffusion in multicomponent compositions.

#### 4.1.2. B-A-H-X Transitions

Calphad calculations correctly predicted B-A transformation in HE-Nd and HE-Gd samples. DTA experiments indicated that transformation proceeds over a 40–70 °C range; however, from Calphad computations, B-A transformation proceeds over a temperature range of several hundred degrees with a change in the fractions and compositions of phases at equilibrium. For HE-Y composition, B-type was predicted to transform directly to the H phase over a narrow biphase region. In contrast, experimental results indicate the B-A transformation in all samples. In agreement with the experiment, the formation of single-phase solid solutions in H and X structure types was predicted for all compositions. H-X transformation was predicted to proceed over an indistinguishably narrow temperature range, with effectively congruent melting at circa ~2400 °C. The narrow biphasic regions on H-X transition and congruent melting from Calphad modeling is consistent with single peaks in DTA.

#### 4.1.3. Biphasic Fields

In a five-component system at constant pressure and temperature, the phase rule allows for up to six phases. In the experiments, we never observed more than two phases at any temperature. Calphad modeling showed mostly single-phase or biphasic fields and very narrow temperature ranges of three-phase co-existence (A, B, C and B, A, H). The absence of large biphasic fields for the B-A transformation from DTA measurements compared with Calphad computations might be attributed to not reaching equilibrium in scanning experiments below 2000 °C. However, the very narrow temperature ranges for H-X transformation and melting are predicted from Calphad and observed in DTA and are likely to be real. In experimental binary phase diagrams of rare earth oxides, the biphasic fields on melting and high temperature transformations are usually not resolved and often added as suggested dotted lines with expectation for them to occur [33,80,81]. Calphad modeling indicates that biphasic fields on A-H-X-L transitions in most binary rare earth oxide systems are often extremely small (~20 °C or less) [67].

It is tempting to assume that observed shrinkage of two-phase fields is the effect of the entropy. Indeed, in many phase diagrams, multiphase fields usually shrink with temperature as solid solution and melt ranges increase. However, that is not always the case. For example, the liquidus loop in the Os-Re system at 3100 °C is as wide as in Cu-Ni system at 1300 °C and shows 20 at.% difference in composition between solid and liquid phases. The narrow two-phase fields for high temperature phase transformations and melting are peculiar to intra-rare earth systems. It was demonstrated in rare earth metal binaries, which melt below 1600 °C and studied more extensively and with higher accuracy than oxide systems [82,83]. Apparent congruent melting across intra-rare earth binaries was discussed in detail by Okatomo and Massalski [83]. Spedding et al. [84] concluded the study of Er-Y and Tb, Dy, Ho, Er binaries with the statement “the isothermal arrests observed for the melting and transformation temperatures show that there is no appreciable enrichment of one component over the other during these processes, which is what would be expected from the general experience encountered in trying to separate rare earths.”

### 4.2. Thermal Expansion and Volume Change on Mixing

A peculiar feature of rare earth oxides is the negative volume change on temperature-induced C-B, B-A, and H-X transformations. In this work, volumes of C- and B-type solid solutions at room temperature were obtained from the analysis of quenched samples, and volumes of B-, A-, H-, and X-type solid solutions were obtained at temperatures of B-A and H-X transitions. This allowed calculation of volume changes on C-B transformation at room temperature, and for B-A and H-X transformation at transition temperatures. Axial thermal expansion of the monoclinic B-type phase was derived from unit cell parameters measured at B-A transition and on samples quenched to room temperature. In the sections below, we compare the behavior of high entropy compositions with pure oxides.

#### 4.2.1. Volume Change on C-B Transition

**Figure 5:**
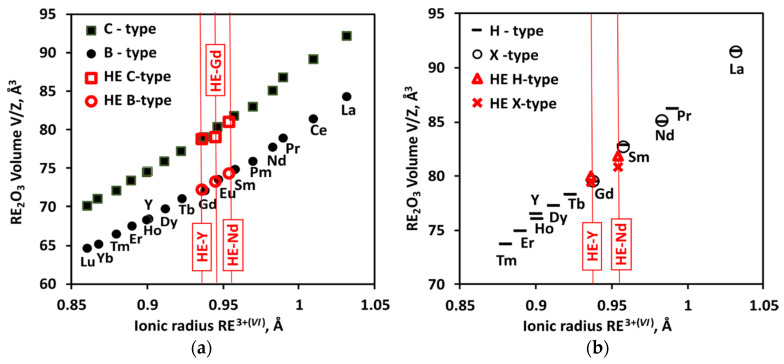
Volumes of C and B phases (**a**) at room temperature and H and X phases (**b**) at transition temperature in pure oxides [30,67] compared with (La_0.2_Sm_0.2_Dy_0.2_Er_0.2_RE_0.2_)_2_O_3_ HE-RE compositions studied in this work, where RE–Nd, Gd, or Y, plotted vs. average ionic radius.

All rare earth sesquioxides can be obtained in their ground-state C-type structure at room temperature. The B-type structure is both a high pressure and a high temperature phase for sesquioxides from Sm to Ho, and high pressure phase for sesquioxides from Er to Lu, Y and Sc [85,86,87,88]. In Figure 5, volumes of C and B phases for samples studied in this work are plotted vs. average ionic radius together with volumes of pure sesquioxides. The volumes of B-type phase in HE samples show no deviation from the trend. For C-type solid solution, only HE-Y sample was obtained as single-phase (in HE-Nd and HE-Gd samples C-type phase coexisted with B-type phase). Nevertheless, the volumes of C-type phase in HE samples show good agreement with the trend.

The volume contraction on C-B transition in HE samples (~8%, Table 2) is indistinguishable from those in constituent oxides, and the B-type phase is both a high temperature and high pressure phase for the studied compositions. This comparison gives no indication of strong deviations from ideal mixing in the solid solution in the B-type structure. For ideal solid solutions, Gibbs energy of mixing does not depend on pressure and there is no excess volume of mixing. For the equiatomic compositions studied in this work, the volume of an ideal solid solution is an average of corresponding volumes of constituent oxides, thus they would follow the trend of volume change vs. average ionic radius.

#### 4.2.2. Thermal Expansion of B-Type Solid Solutions

Taylor [89] reviewed thermal expansion data for pure rare earth sesquioxides and found no data on axial expansion for B-type phases. Ploetz et al. [90] studied the linear expansion of B-type Gd, Eu, and Sm sesquioxides by interferometry and reported the values (10.0–10.8) × 10^−6^ /K for 30 to 850 °C range. Since the Ploetz et al. measurements were performed on polycrystalline samples, volumetric thermal expansion can be estimated as three times the linear expansion and corresponds to (3.0–3.2) × 10^−5^/K. These values are in good agreement with average volume thermal expansion for multicomponent rare earth oxides measured in this work: (3.5 ± 0.2) × 10^−5^/K from room temperature to the B-A transformation temperatures (1916–1975 °C).

#### 4.2.3. Volume Changes on B-A and H-X Transformations

The volume change on B-A transformation was refined for HE-Nd and HE-Gd samples as −2.5 ± 0.1% and for HE-Y sample as −3.1 ± 0.1% The volume change on H-X transformation was refined for He-Nd and HE-Y samples as −1.2 ± 0.1 and −0.6 ± 0.1%, respectively. Since A, H, and X phases were not quenchable in these compositions, our values were determined from diffraction experiments at the respective transition temperatures. The volume change on B-A and H-X transformation reported for pure oxides are about −2% and −0.5%, respectively [30,67]. Thus, within the resolution of the data, we do not observe any anomalies in volume change on B-A, and H-X transformation in the studied solid solutions compared with pure oxides.

#### 4.2.4. Volumes of H-Type Solid Solution vs. Pure Oxides

In Figure 5, the volumes of the H-type phase for HE-Nd and HE-Y are overlaid with values reported by Foex and Traverse [30] for pure oxides. Foex and Traverse’s values correspond to the temperatures from 2120 °C for La_2_O_3_ to 2330 °C for Ho_2_O_3_ and Y_2_O_3_. The volume for the H phase for Sm_2_O_3_ and Gd_2_O_3_ refers to the temperatures 2200 and 2250 °C, respectively. Our data on the volume of H phase in HE-Nd and He-Gd from diffraction on levitated samples were assigned to temperatures 2202 ± 4 °C and 2254 ± 8 °C based on co-existence with X-type phase (Table 5) and DTA results. Thus, we can compare volumes of solid solutions vs. pure oxides at a similar temperature. The observed agreement in volume is within 2%. In contrast with the calculation of volume change on transition in coexisting phases, XRD measurement of the absolute values for the volumes at high temperature are affected by sample shift and temperature calibration. Thus, within the resolution of the data, we do not observe deviations from ideal volumetric mixing in the H-type solid solution for the studied compositions.

### 4.3. Increase of Melting Temperature in HE-RE

The measured melting temperature for HE-Nd is 2456 ± 12 °C. The error is assigned based on uncertainty in melting temperature of Y_2_O_3_ used for calibration, 2439 ± 12 °C [73]. If the solid solution were to melt congruently, one might estimate, to a very crude first approximation, its melting point as a weighted average of the endmember melting points [91], namely 2357 ± 16 °C. The uncertainties assigned to melting points of constituent oxides in HE-Nd (La,Sm,Dy,Er,Nd)_2_O_3_ composition are similar to Y_2_O_3_ (±15 vs. ±12 °C), however, Y_2_O_3_ melting temperature was established from independent measurements in ten laboratories [73], while values for Dy_2_O_3_ and Er_2_O_3_ are based on results of one group and regarded only as “probable” [91].

This observation of apparent increase of melting temperature compare with constituent oxides may be attributed to a combination of kinetic and thermodynamic factors. If the observed behavior does represent equilibrium, then the solidus–liquidus relations must be strongly perturbed by nonideal mixing behavior in the solid, liquid, or both.

To raise the melting temperature, nonideality must favor the solid over the liquid. This can occur by one or both of the following: negative deviations from ideality in the solid phase or positive deviations in the liquid phase. Negative deviations in the solid solution are generally associated with local ordering, hinting at a tendency toward compound formation, and resulting in negative heats of mixing. The volumetric behavior described above shows no evidence for ordering, but volume is not necessarily a good proxy for energetics. The other possibility is strongly positive deviations from ideal mixing in the molten oxides, leading, in the extreme, to liquid–liquid phase separation. We know very little about the structure and thermodynamics of molten binary or multicomponent rare earth oxide systems, except for a recent report from Nakanishi and Allanore [92] on non-ideal mixing in La_2_O_3_-Y_2_O_3_ melt, so this possible scenario cannot be assessed at present.

Increases in melting temperature in multicomponent systems compared with end members is not common in oxides, however, it is known to occur, most notably on the ZrO_2_-rich side of binaries with Dy-Yb and Y sesquioxides, where melting temperature increase over 100 °C compared with pure ZrO_2_ for the solid solution with 25 mol. % of Yb_2_O_3_ [93]. This almost certainly relates to the formation of favorable short-range order and defect clustering involving tetravalent and trivalent ions and oxygen vacancies. Such a short-range order probably is less important in the trivalent oxide systems studied here. In our system, it is probable that the melting temperature increase should be manifested at least in one of the related binary systems. No increase in melting temperatures was reported in studied binaries with La_2_O_3_ [33,94,95]. Sm, Dy, and Er are a common constituent in all HE compositions studied in this work. The study of liquidus in the binary systems, currently unknown, is needed to further identify the extent and source of the increase of melting temperatures observed in HE-RE compositions.

## 5. Conclusions

In this work, we studied three five-component compositions of trivalent rare earth sesquioxides: (La,Sm,Dy,Er,RE)_2_O_3_, with all oxides in equimolar ratios and RE either Nd or Gd or Y. All studied compositions demonstrated a C-B-A-H-X transformation sequence into structure types typical for rare earth sesquioxides. Monoclinic B-type phase was obtained in all compositions by laser melting and splat quenching and was retained after prolonged annealing at 800 °C. The experimentally observed phases are in good agreement with Calphad calculations performed using thermodynamic data for pure sesquioxides and the ideal solution model.

Compared with constituent oxides, A-type and X-type phases occur at wider temperature ranges in the studied compositions. The measured room temperature volumes of C and B phases and volume changes on C-B, B-A, and H-X transitions are in good agreement with those predicted from constituent oxides. No anomalies in thermal expansion of B-type solid solution and in volumes of H-type phases were detected. The obtained data on temperatures, enthalpies, and entropies of transitions can be used for benchmarking the next generation of thermodynamic databases for rare earth oxides. The observed increase in melting temperature compared with constituent oxides invites experimental and theoretical investigations of Sm_2_O_3_-Dy_2_O_3_, Dy_2_O_3_-Er_2_O_3_, Er_2_O_3_-Sm_2_O_3_ systems for which no data on melting temperatures are available.

## Figures and Tables

**Table 1:** Atomic percent of rare earth cations in laser-melted rare earth sesquioxides from the results of wavelength dispersive microprobe analysis.

Rare Earth	HE-Nd	HE-Gd	HE-Y
La	19.5 ± 0.2	18.7 ± 0.4	17.7 ± 0.8
Sm	20.1 ± 0.1	20.7 ± 0.1	19.8 ± 0.1
Dy	20.8 ± 0.1	21.1 ± 0.2	18.0 ± 0.3
Er	20.3 ± 0.1	20.3 ± 0.2	18.4 ± 0.3
Y	-	-	26.0 ± 0.4
Gd	-	19.3 ± 0.2	-
Nd	19.3 ± 0.1	-	-
MW g/mol	350.89	358.84	323.09
Ave radii ^1^ Å	0.954	0.945	0.936

^1^ The average ionic radius of rare earth after Shannon [71] for RE^+3^ in octahedral coordination.

**Table 2:** The phase composition of powders after calcination at 800 °C and heat treatment at 1100 °C.

Experiment	Phase	HE-Nd	HE-Gd	HE-Y
Air 800 °C 96 h	**C-Type (Cubic, Bixbyite-Type) *Ia*-3, Z = 16**			
	*a*, Å	10.903(1)	10.863(2)	10.814(3)
	V, A^3^/z	81.0(1)	80.1(1)	79.0(1)
	Size	38 ± 1 nm	21 ± 1 nm	21 ± 1 nm
	wt.%	~85 wt.%	~90 wt.%	100 wt.%
	**B-Type (Monoclinic, Sm_2_O_3_-Type) *C*2/*m*, Z = 6**			
	*a*, Å	14.259(5)	13.90(4)	-
	*b*, Å	3.620(1)	3.53(5)	
	*c*, Å	8.862(3)	9.00(4)	
	β, °	100.67(1)	96.8(1)	
	V, A^3^/z	74.9 ± 0.1	73 ± 2	
	Size	32 ± 2 nm	13 ± 2 nm	
	wt.%	~15 wt.%	~10 wt.%	
	V (C→B) %	−7.5 ± 0.1%	−9.7 ± 0.3%	
Air 1100 °C 12 h	**C-type (cubic, bixbyite-type) *Ia*-3, Z = 16**			
	*a*, Å		10.825(1)	10.804(1)
	V, A^3^/z	-	79.0(1)	78.8(1)
	Size		49 ±1 nm	65 ± 1 nm
	wt.%		~30 wt.%	100 wt.%
	**B-type (monoclinic, Sm_2_O_3_-type) *C*2/*m*, Z = 6**			
	*a*, Å	14.242(1)	14.227(1)	
	*b*, Å	3.6152(2)	3.601(1)	
	*c*, Å	8.857(1)	8.833(1)	-
	β, °	100.62(4)	100.66(1)	
	V, A^3^/z	74.7(1)	74.1(1)	
	Size	73 ± 1 nm	68 ± 1 nm	
	wt.%		~70 wt.%	
	V (C→B) %	−8.7 ± 0.1%	−6.8 ± 0.1%	

The numbers in brackets indicate uncertainty in the last digit from the refinement of XRD data.

**Table 3:** Room temperature unit cell parameters and crystallite sizes of HE samples after splat quenching from ~3000 °C, and after laser melting and annealing at 800 °C for 60 days. All samples were indexed in a monoclinic B-type structure (S.G. *C*2*/m*, Sm_2_O_3_-type).

Unit Cell	HE-Nd *		HE-Gd		HE-Y	
Parameters	Splat Quench	800 °C/60 d	Splat Quench	800 °C/60 d	Splat Quench	800 °C/60 d
*a*, Å	14.245(1)	14.244(1)	14.194(1)	14.180(1)	14.159(2)	14.139(1)
*b*, Å	3.6150(1)	3.6025(1)	3.5956(1)	3.5840(1)	3.5741(2)	3.5617(1)
*c*, Å	8.857(1)	8.839(1)	8.818(1)	8.797(1)	8.781(1)	8.758(1)
β, °	100.63(1)	100.69(1)	100.59(1)	100.59(1)	100.61(1)	100.65(1)
V, A^3^/z	74.72(1)	74.28(1)	73.73(1)	73.24(1)	72.79(1)	72.24(1)
Cryst. size	76 ± 1 nm	95 ± 1 nm	80 ± 1 nm	101 ± 1 nm	86 ± 1 nm	147 ± 3 nm

* Cell parameters refined with internal standard on HE-Nd sample after HTXRD experiments (*a*, *b*, *c*, β): 14.244(1) Å, 3.610(2) Å, 8.852(2) Å, 100.611(5)°.

**Table 4:** Results of thermal analysis of (La_0.20_Sm_0.20_Dy_0.21_Er_0.20_Nd_0.19_)_2_O_3_ (HE-Nd), (La_0.19_Sm_0.21_Dy_0.21_Er_0.20_Gd_0.19_)_2_O_3_ (HE-Gd)_,_ and (La_0.18_Sm_0.20_Dy_0.18_Er_0.18_Y_0.26_)_2_O_3_ (HE-Y) samples.

	HE-Nd	HE-Gd	HE-Y
T_B-A_ °C	1916 ± 9(5) *	1957 ± 5(4)	1975 ± 13(4)
H_B-A_ J/g	57 ± 3(10)	56 ± 7(8)	56 ± 8(8)
H_B-A_ kJ/mol	19.8 ± 1.0	20.3 ± 2.7	18.0 ± 2.7
S_B-A_ J/mol/K	9.0 ± 0.1	9.1 ± 0.1	8.0 ± 0.2
T_A-H_ °C	2125 ± 3(5)	2180 ± 2(4)	2199 ± 4(6)
H_A-H_ J/g	22 ± 3(10)	23 ± 1(7)	28 ± 1(9)
H_A-H_ kJ/mol	7.7 ± 0.9	8.3 ± 0.5	9.2 ± 0.3
S_A-H_ J/mol/K	3.2 ± 0.1	3.4 ± 0.1	3.7 ± 0.1
T_H-X_ °C	2202 ± 4(2)	2235 ± 5(2)	2254 ± 8(4)
H_H-X_ J/g	79 ± 1(2)	85 ± 23(3)	126 ± 7(4)
H_H-X_ kJ/mol	27.8 ± 0.2	30.6 ± 8.3	40.6 ± 2.4
S_H-X_ J/mol/K	11.2 ± 0.1	12.2 ± 0.4	16.1 ± 0.1
T_m_ °C	2456 ± 12		

* The uncertainties are reported as two standard deviations of the mean with the number of experiments given in parentheses.

**Table 5:** High-temperature unit cell parameters and volume changes on B-A and H-X transformation in HE samples from synchrotron diffraction on laser-heated levitated samples.

Structure/Sample		HE-Nd	HE-Gd	HE-Y
T_tr_ (DTA)	T_B-A_, °C	1916 ± 9	1957 ± 5	1975 ± 13
B-type monoclinic *C*2/*m*, Z = 6	*a*, Å	14.433(8)	14.403(5)	14.367(7)
	*b*, Å	3.711(1)	3.697(1)	3.674(1)
	*c*, Å	9.026(5)	8.987(2)	8.965(3)
	β, °	101.35(2)	101.18(1)	101.09(2)
	V, A^3^/z	79.0(2)	78.2(1)	77.4(1)
A-type trigonal *P*-3*m*1, Z = 1	*a*, Å	3.885(1)	3.874(1)	3.864(1)
	*c*, Å	6.199(1)	6.175(2)	6.171(1)
	V, A^3^/z	81.03(2)	80.24(2)	79.78(1)
	V (B→A), %	−2.5 ± 0.1	−2.5 ± 0.1	−3.1 ± 0.1
**T_tr_ (DTA)**	**T_H-X_, °C**	**2202 ± 4**	**2235 ± 5**	**2254 ± 8**
H-type *P*63/*mmc*, Z = 1	*a*, Å	3.898(1)		3.869(1)
	*c*, Å	6.216(1)		6.168(1)
	V, A^3^/z	81.81(1)		79.96(1)
X-type *Im*-3*m*, Z = 1	*a*, Å	4.324(1)		4.2989(1)
	V, A^3^/z	80.85(2)		79.44(1)
	V (H→X), %	−1.2 ± 0.1		−0.6 ± 0.1

## Supplementary Materials

The following are available online at https://www.mdpi.com/1996-1944/13/14/3141/s1, Figure S1: The flow chart of performed experiments, Figure S2: Aerodynamic levitator with splittable nozzle and copper plates for splat quenching. Figure S3: Integration of area detector diffraction images of aerodynamically levitated bead, Figures S4–S6: Back-scattered electron micrograph of the laser-melted samples, Figure S7: Room-temperature X-ray diffraction patterns of HE-Y, HE-Gd and HE-Nd samples from solution combustion synthesis, Figure S8: Rietveld refinement plot of HE-Nd sample after calcination in air at 800 °C for 96 h, Figure S9: Rietveld refinement plot of HE-Nd sample after splat quenching from melt, Figure S10: Room-temperature powder XRD patterns on Sm_2_O_3_ sample after annealing at 800 °C and after laser melting, Figure S11: Heat flow trace vs. sample temperature for HE-Gd sample, Figure S12: Pawley refinement of unit cell parameters for B and A phases of HE-Gd sample at transition temperature, Figure S13: Pawley refinement of unit cell parameters for H and X phases of HE-Y sample at transition temperature, Figure S14: Calphad modeling of phase fractions in HE-Y, HE-Gd and HE-Nd samples.
