# Supplementary material for: Thermal Analysis of High Entropy Rare Earth Oxides
Source: Materials (Basel). 2020 Jul 14;13(14):3141. doi: 10.3390/ma13143141 (PMC7412006; doi:10.3390/ma13143141)
Supplement: Supplementary file 1 [file materials-13-03141-s001.pdf]

materials

Supporting Information

Thermal Analysis of High-Entropy Rare Earth Oxides

Sergey V. Ushakov [1,*], Shmuel Hayun [2,*], Weiping Gong [3,*] and Alexandra Navrotsky [1,*]

1. School of Molecular Sciences, and center for Materials of the Universe, Arizona State University, Tempe, AZ 85287, USA
2. Department of Materials Engineering at the Ben-Gurion University of the Negev, Beer-Sheva 84105, Israel
3. Guangdong Provincial Key Laboratory of Electronic Functional Materials and Devices, Huizhou University, Huizhou 516001, Guangdong, P. R. China

* Correspondence: sushakov@asu.edu (S.V.U.); hayuns@bgu.ac.il (S.H.); weiping_gong@csu.edu.cn (W.G.); anavrots@asu.edu (A.N.)

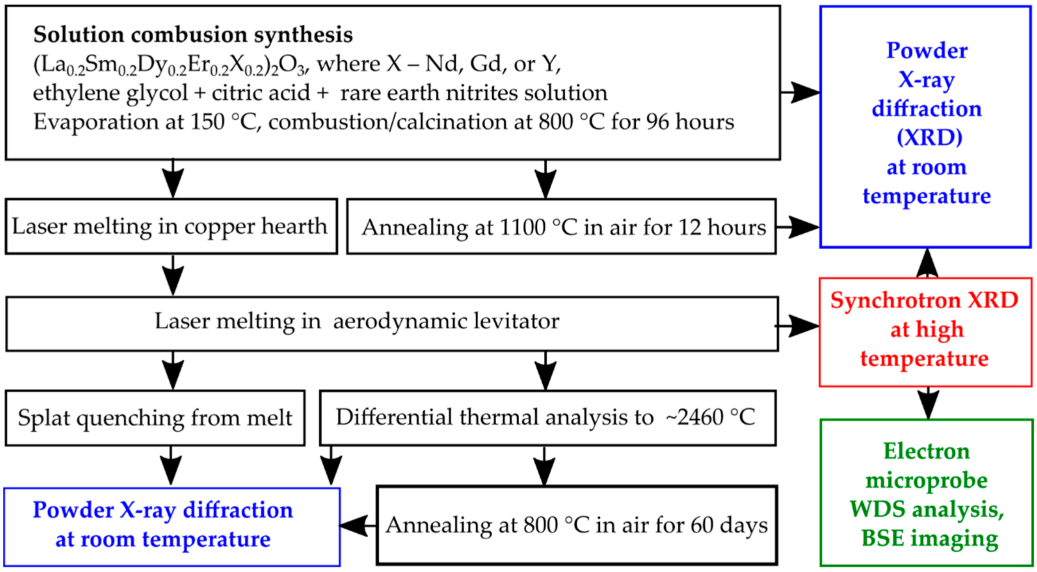

Figure S1. The flow chart of the performed experiments and characterization

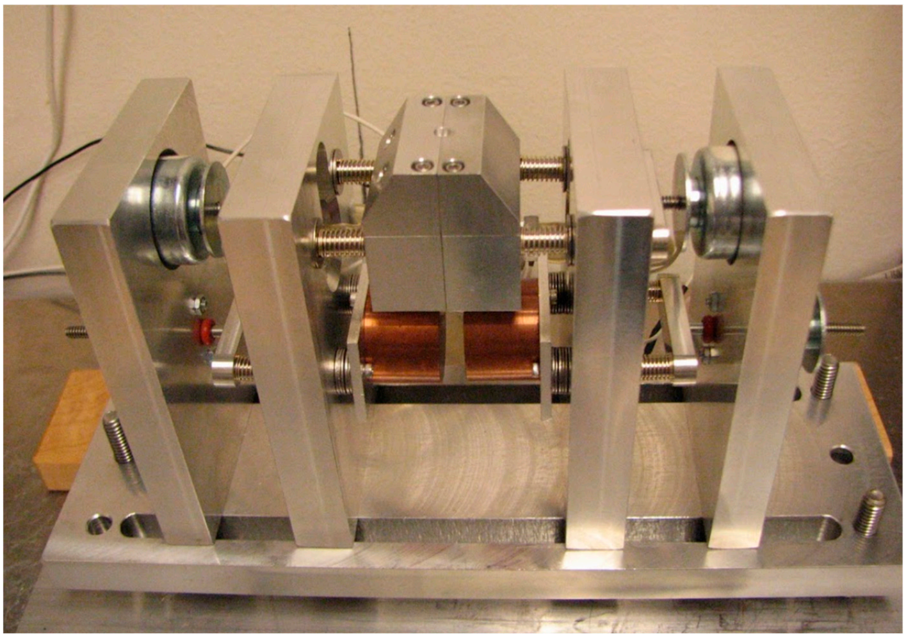

Figure S2. Aerodynamic levitator with splittable nozzle and copper plates for splat quenching. The outer diameter of the nozzle is 5 mm. Sample is heated from the top with 400-W CO_2 laser.

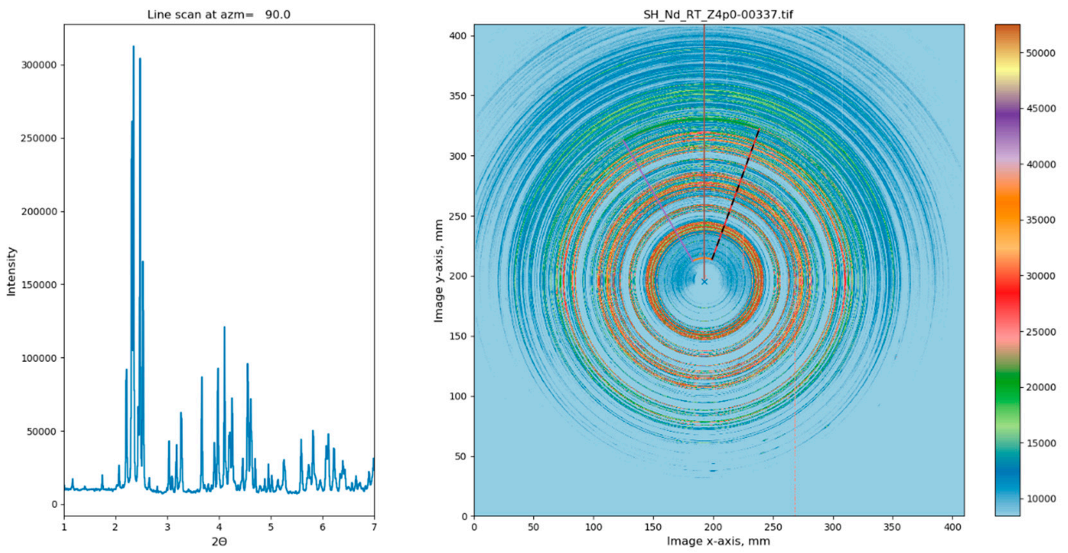

Figure S3. Integration of area detector diffraction images of aerodynamically levitated bead at 6-ID-D beamline of Advanced Phton Source (APS). Synchrotron X-ray wavelength λ = 0.123613 Å. The shown image is a sum of 100 images collected with acquisition interval 0.1s on HE-Nd (($La_{0.20}Sm_{0.20}Dy_{0.21}Er_{0.20}Nd_{0.19})_2O_3$) bead levitated at room temperature in Ar flow. The intensities in the lower part of the image are attenuated by a levitation nozzle. The integration settings used were as follows: inner/outer 2-theta; 1.0–7.0°; start/end azimuth 70.0–120.0°; 1600 2-theta steps).

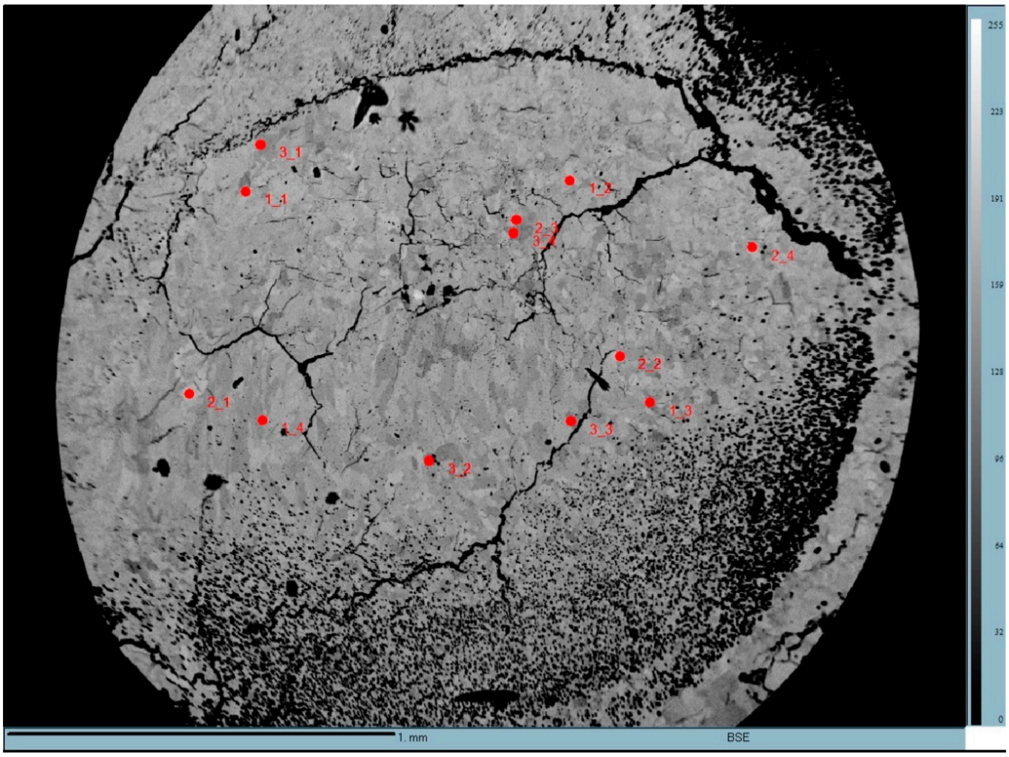

Figure S4. Back-scattered electron micrograph of the laser-melted HE-Y (($La_{0.18}Sm_{0.20}Dy_{0.18}Er_{0.18}\mathbf{Y_{0.26}})_2O_3$)) sample with points of microprobe analysis.

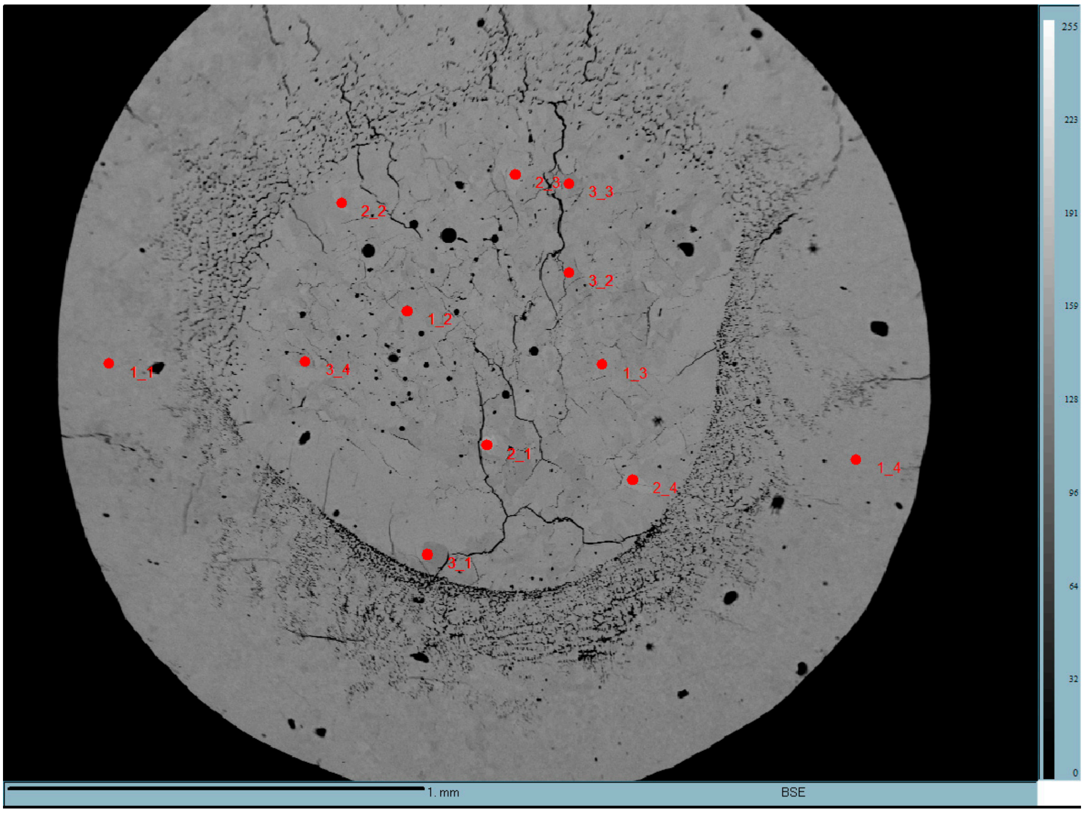

Figure S5. Back-scattered electron micrograph of the laser-melted HE-Gd (($La_{0.19}Sm_{0.21}Dy_{0.21}Er_{0.20}Gd_{0.19})_2O_3$) sample with points of microprobe analysis.

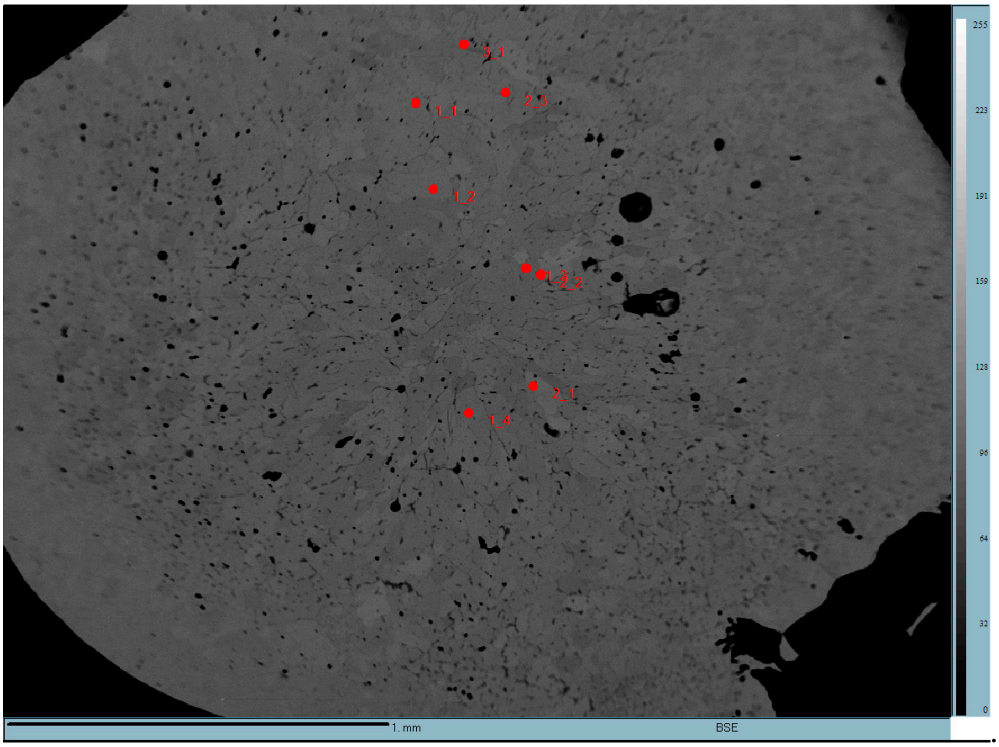

Figure S6. Back-scattered electron micrograph of the laser-melted HE-Nd (($La_{0.20}Sm_{0.20}Dy_{0.21}Er_{0.20}Nd_{0.19})_2O_3$) sample with points of microprobe analysis.

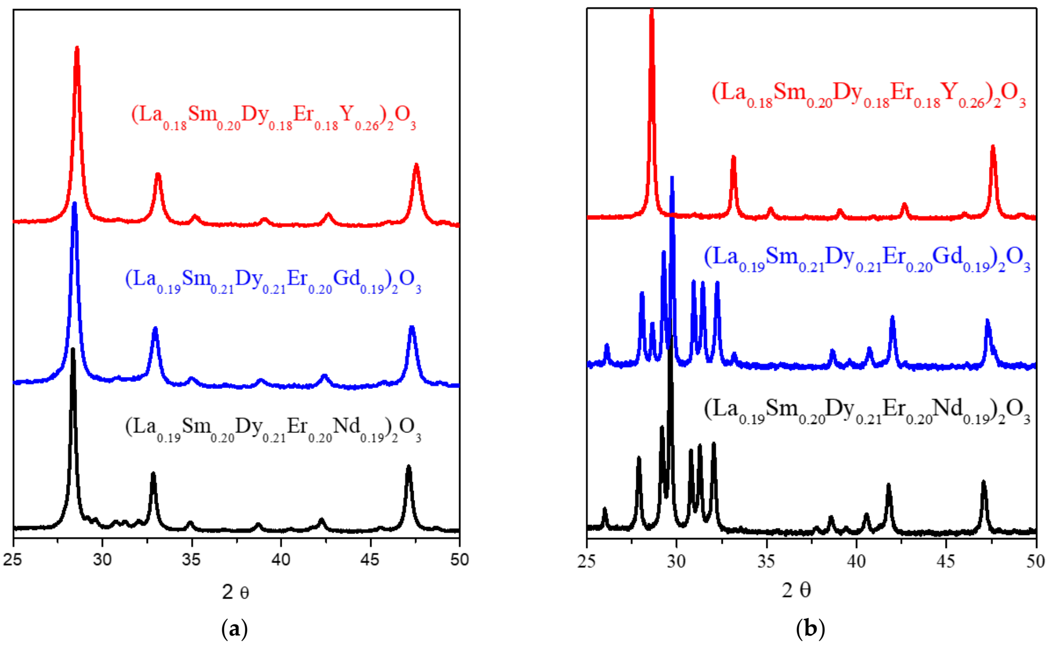

Figure S7. Room-temperature X-ray diffraction patterns of HE-Y, HE-Gd and HE-Nd samples from solution combustion synthesis: (**a**) After calicination at 800 °C for 96 h; (**b**) after annealing at 1100 °C for 12 h (Cu Ka radiation λ = 1.54056 Å).

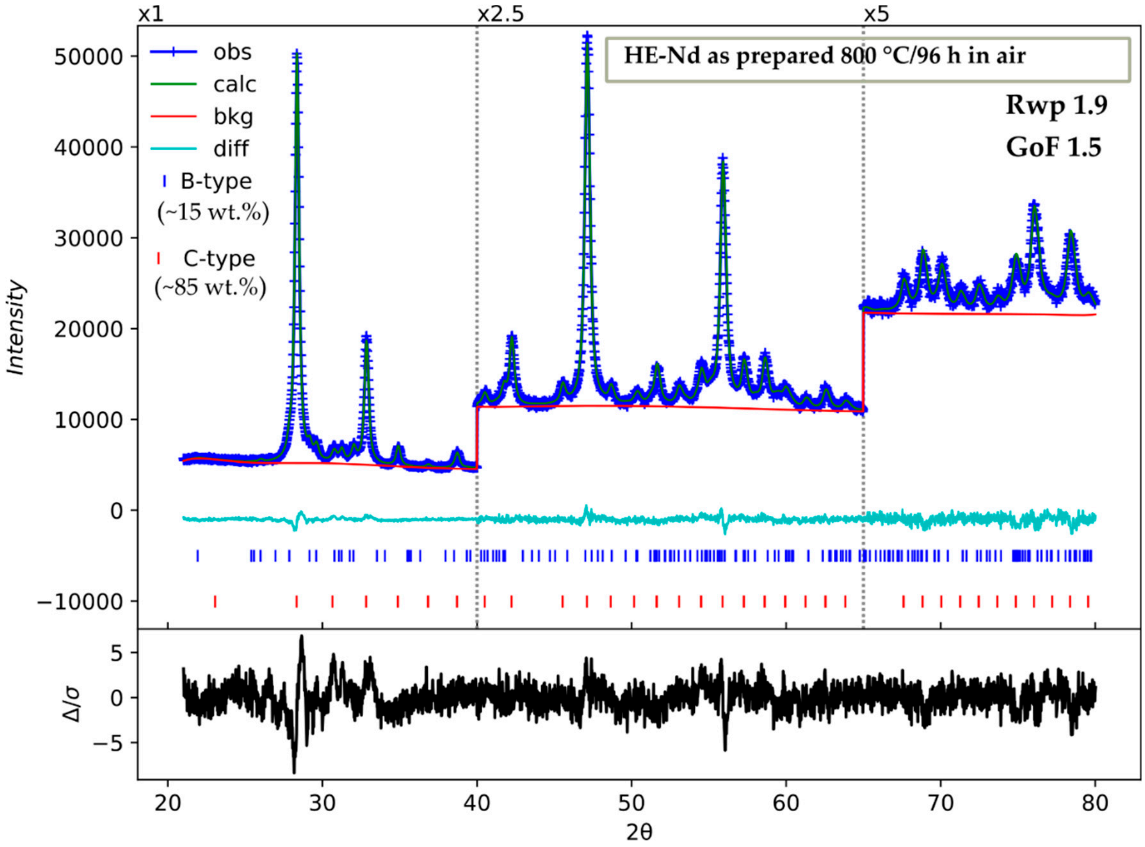

Figure S8. Rietveld refinement plot of HE-Nd ($(La_{0.20}Sm_{0.20}Dy_{0.21}Er_{0.20}Nd_{0.19})_2O_3$) sample after calcination in air at 800 °C for 96 h. Powder X-ray diffraction pattern was collected at room temperature using Cu Kα radiation λ = 1.54056 Å.

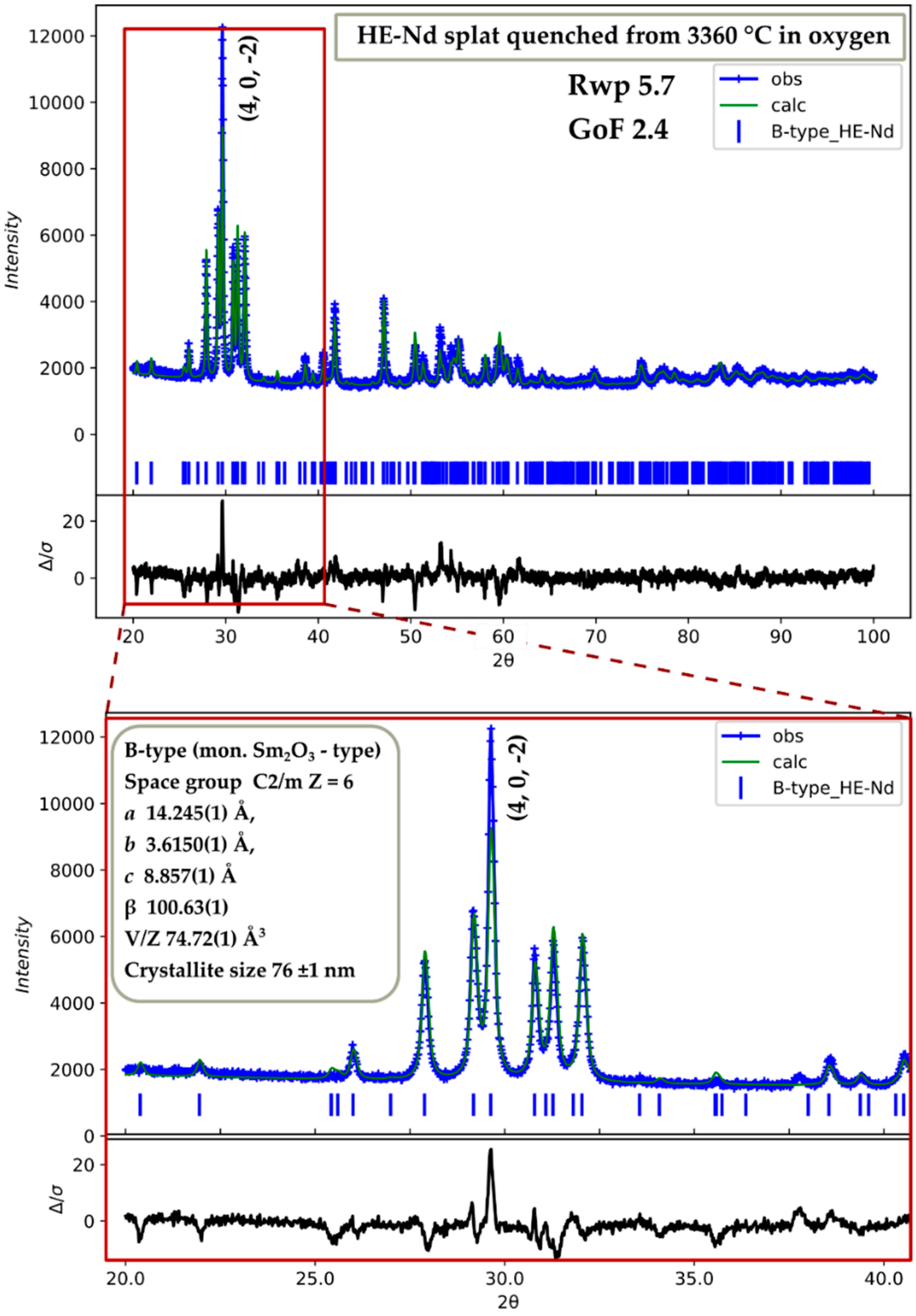

Figure S9. Rietveld refinement plot of HE-Nd ($(La_{0.20}Sm_{0.20}Dy_{0.21}Er_{0.20}Nd_{0.19})_2O_3$) sample after splat quenching from melt. Powder X-ray diffraction pattern was collected at room temperature using Cu Kα radiation $\lambda = 1.54056$ Å.

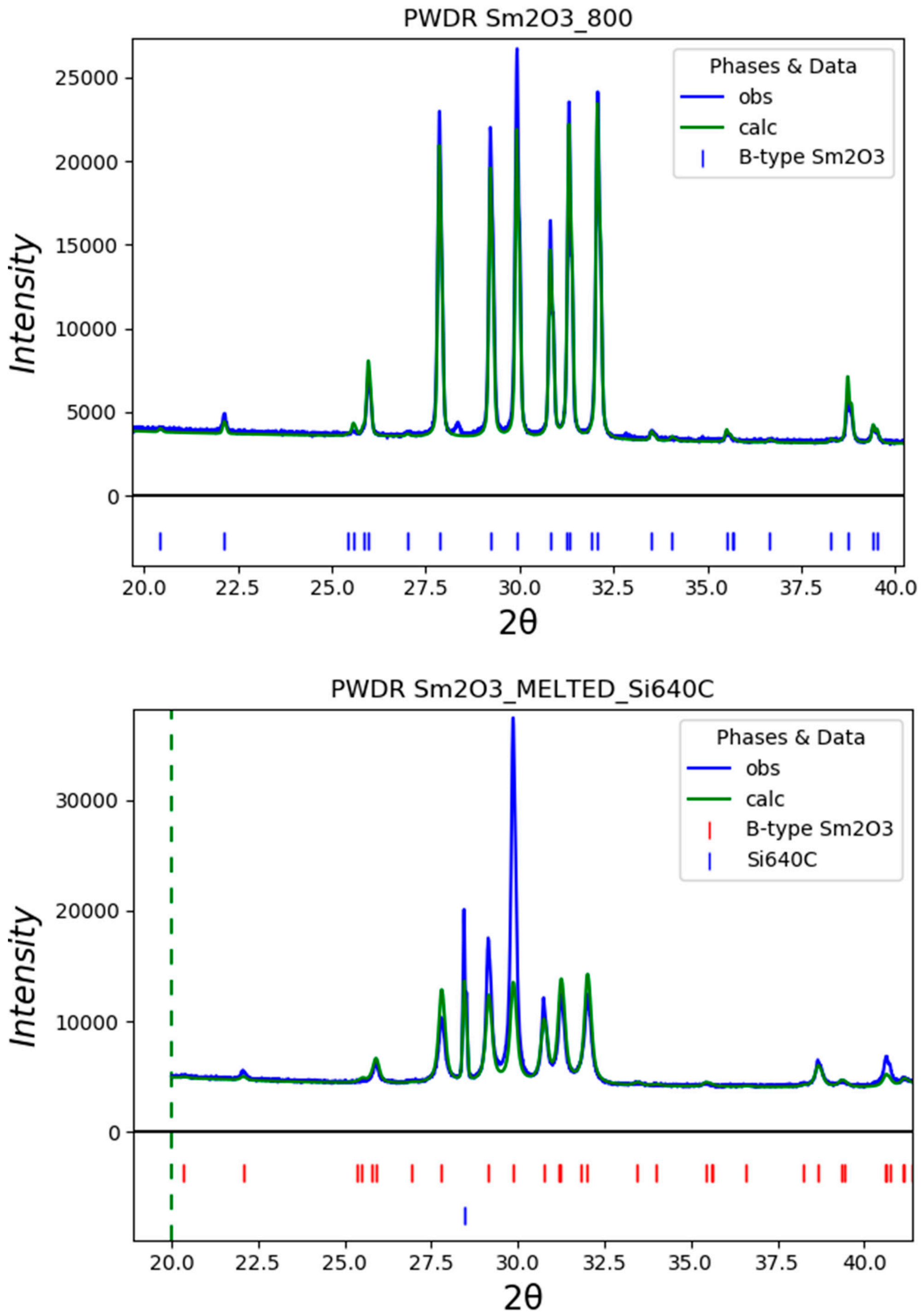

Figure S10. Room-temperature powder XRD patterns on Sm_2O_3 sample (Alfa Aesar 99.99% purity) after annealing at 800 °C (top) and after laser melting (bottom). (4, 0, −2) reflection is marked. NIST Si640C standard was added in the sample after laser melting.

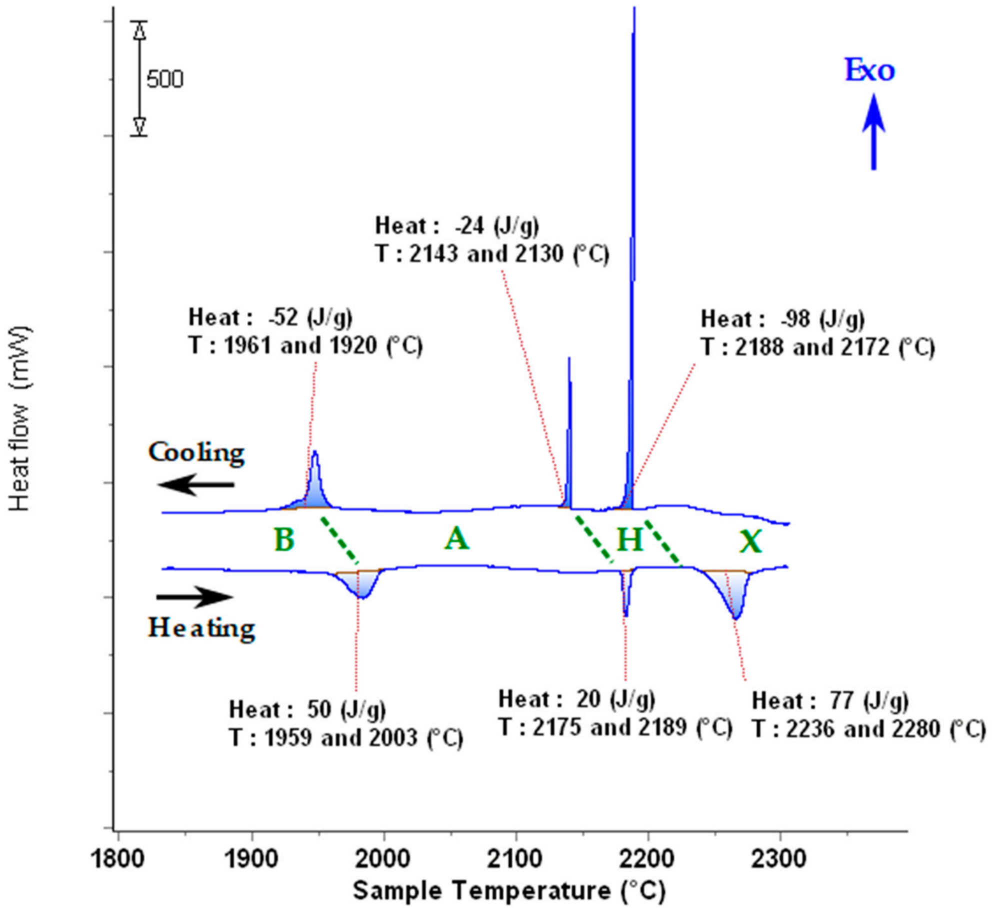

Figure S11. Heat flow trace (baseline subtracted) vs. sample temperature for HE-Gd ($(La_{0.19}Sm_{0.21}Dy_{0.21}Er_{0.20}Gd_{0.19})_2O_3$) sample. Sample mass 140.23 mg. Heating and cooling rate 20 °C/min. Three endothermic peaks on heating and corresponding exothermic peaks on cooling are related to reversible B-A, A-H and H-X transformations. Temperatures corresponding to onset of the transition and to return to the baseline are labelled for each peak.

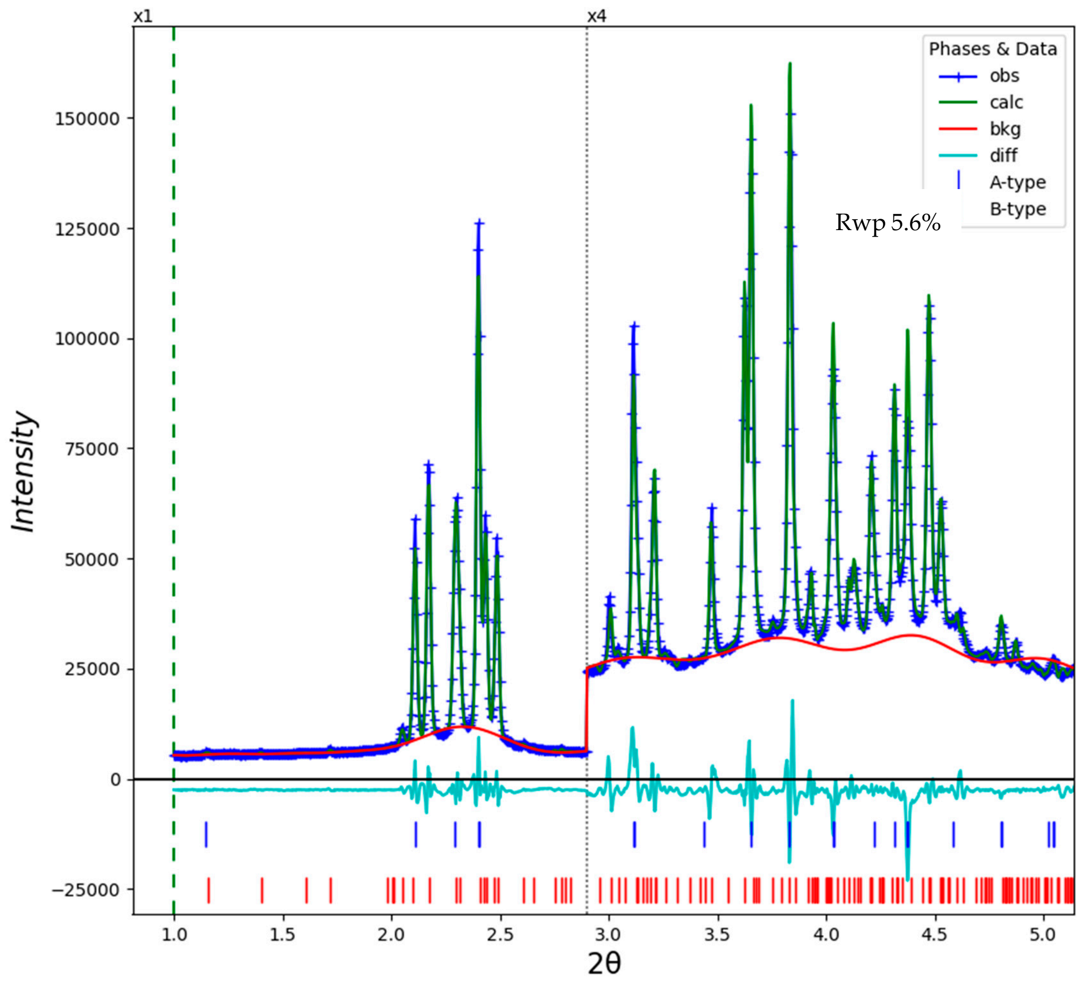

Figure S12. Pawley refinement of unit cell parameters for B and A phases of HE-Gd sample at transition temperature (1957 ± 10 °C from DTA results). XRD pattern collected on laser-heated sample aerodynamically levitated in argon, X-ray wavelength λ = 0.1236 Å.

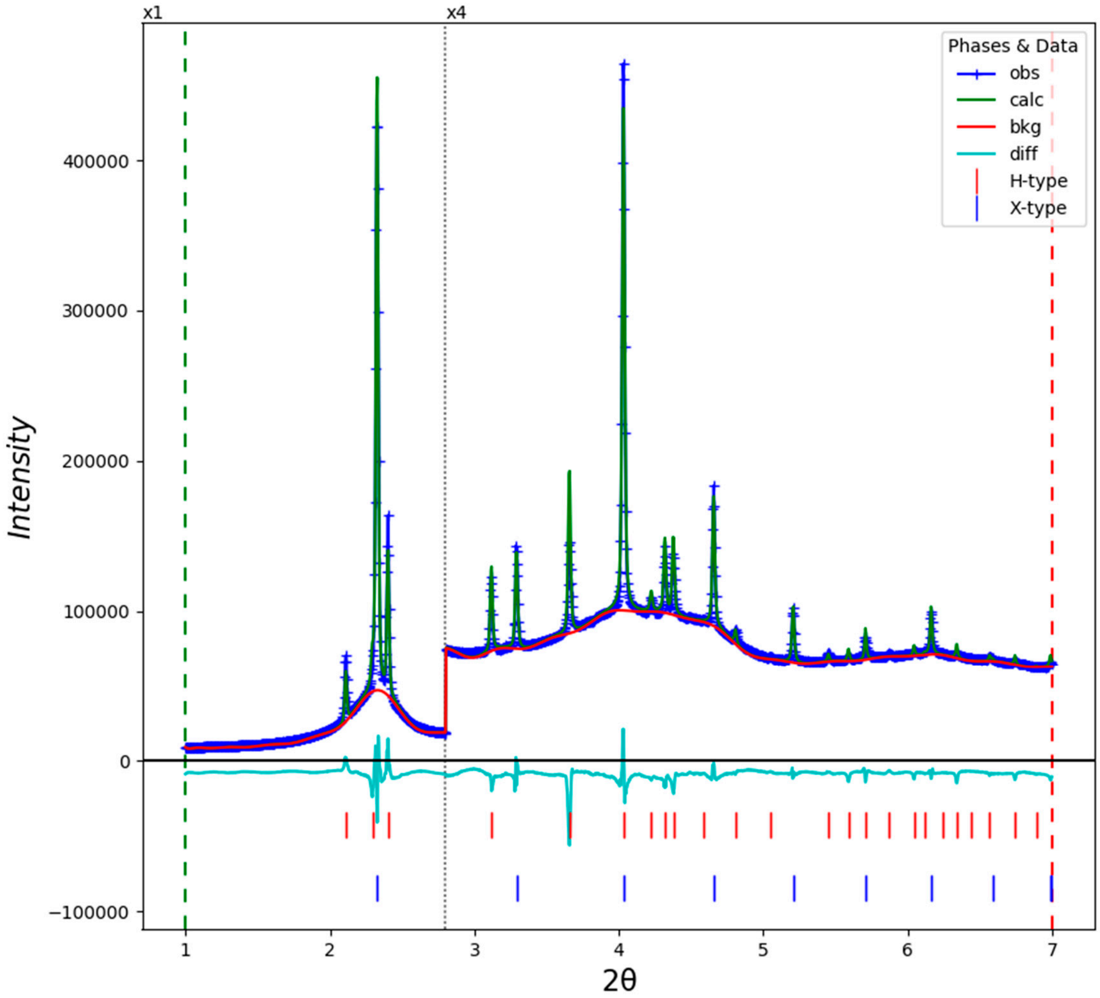

Figure S13. Pawley refinement of unit cell parameters for H and X phases of HE-Y sample at transition temperature (2254 ± 8 °C from DTA results). XRD pattern collected on laser-heated sample aerodynamically levitated in argon, X-ray wavelength λ = 0.1236 Å.

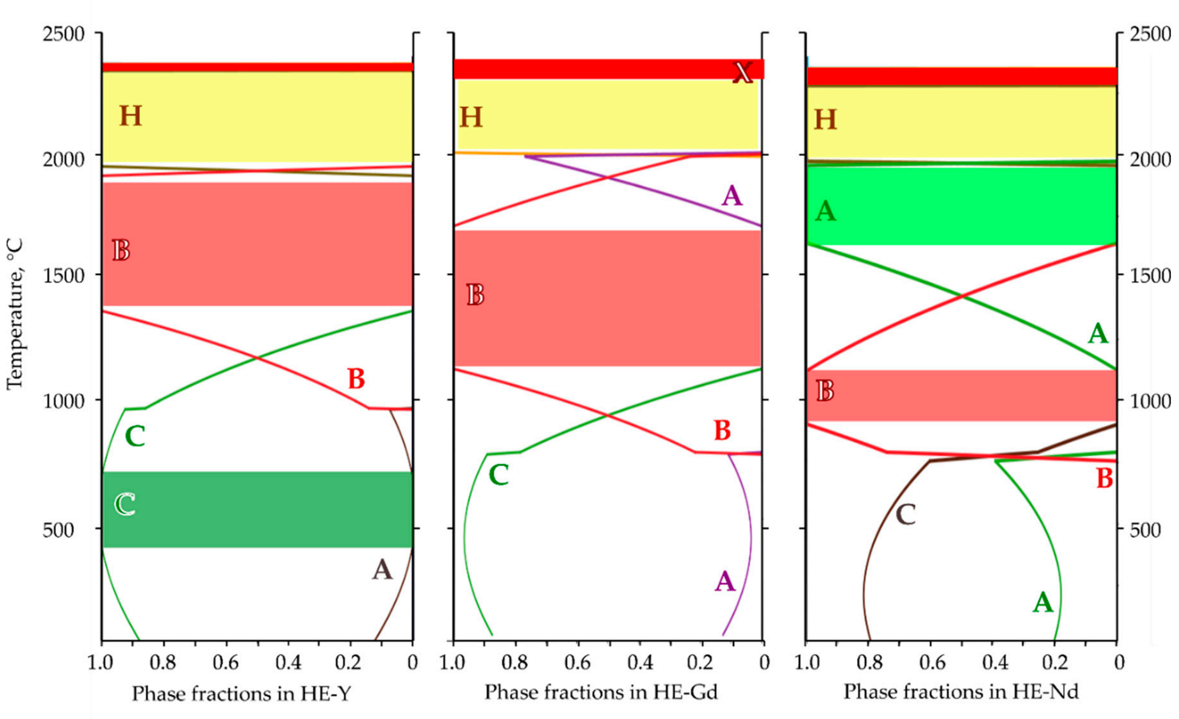

Figure S14. Calphad modeling of phase fractions in HE-Y ($(La_{0.18}Sm_{0.20}Dy_{0.18}Er_{0.18}Y_{0.26})_2O_3)$), HE-Gd ($(La_{0.19}Sm_{0.21}Dy_{0.21}Er_{0.20}Gd_{0.19})_2O_3$) and HE-Nd ($(La_{0.20}Sm_{0.20}Dy_{0.21}Er_{0.20}Nd_{0.19})_2O_3$) samples. The single phase fields are shaded.

© 2020 by the authors. Submitted for possible open access publication under the terms and conditions of the Creative Commons Attribution (CC BY) license (http://creativecommons.org/licenses/by/4.0/).
